# Neurosyphilis in AIDS patient presenting as isolated cranial nerve 6 palsy

**DOI:** 10.1002/ccr3.3018

**Published:** 2020-06-26

**Authors:** Sanjay Singh, Priyangi Puranik, Ethan Lin, Miriam Marti, Antonio Liu

**Affiliations:** ^1^ Ross School of Medicine Bridgetown Barbados; ^2^ Department of Family Practice California Hospital Medical Center Los Angeles California USA; ^3^ Department of Neurology Adventist Health White Memorial Los Angeles California USA

**Keywords:** HIV, infectious disease, neurology, neurosyphilis, syphilis

## Abstract

Early neurosyphilis can occur in an immunocompromised host. It has a widely varied presentation. Isolated CN6 as presenting symptom has not been described.

## Graphical abstract

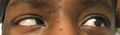

## 1 INTRODUCTION

Syphilis is caused by an infection with *Treponema pallidum* which is sexually transmitted. Neurosyphilis is the tertiary stage of the infection, and it causes multiple, variable, and complex presentation within the central nervous system (CNS). There are numerous nonspecific manifestations including stroke‐like symptoms, hemiparesis, sudden blindness, and optic neuritis.^1^, ^2^, ^3^, ^4^ Due to a wide range of clinical symptoms, establishing the diagnosis and initiating treatment in a timely manner can be challenging. Cerebrospinal fluid (CSF) findings of elevated protein and lymphocyte count with positive venereal disease research laboratory test (VDRL) suggest neurosyphilis. Neurosyphilis usually presents many years after initial infection in immunocompetent host. However, in human immunodeficiency virus (HIV)‐infected patients, the symptoms appear much earlier than expected.^5^, ^6^, ^7^ We present a 34 years old HIV‐positive African American man who presented with diplopia caused by isolated left cranial nerve (CN) 6 palsy. He was diagnosed with neurosyphilis with CSF analysis.

## 2 CASE

A 34‐year‐old man with AIDS came to Emergency Department with 3 days of diplopia. Two months prior to this visit, he was admitted for headache and mild altered mental status. CT and MRI brain were negative. His peripheral blood rapid plasma reagin (RPR) test was positive with a titer of 1:8. His fluorescent treponemal antibody absorption test (FTA‐ABS) was positive. He was found to have neurosyphilis as cerebrospinal fluid analysis showed VDRL at 1:16 titer. CSF white blood cell count (WBC) was 45 cell/cubic mm, 95% lymph. CSF protein was 50 mg/dL, and glucose was 39 mg/dL. His maximum temperature was 39.9℃ during that hospitalization. There was no focal neurological finding on examination. Antibiotics and highly active antiretroviral therapy (HAART) were initiated; however, he left the hospital against our advice after 4 days of treatment.

**Figure 1:**
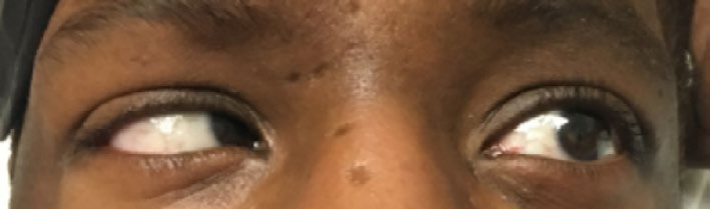
Inability to fully abduct left eye while looking toward the left

On this current admission, patient stated that he had been well until 3 days ago. He has recurrent headaches and experienced double vision upon looking to the left. He admitted that he was not adhering to antibiotics or HAART. He was found to be coherent, noncachectic with stable vital signs. He was alert and oriented to name, place, time, and situation. There were no headaches, and neck was supple. Cranial nerve 2‐12 were intact except the isolated left CN 6 partial palsy. He partially abducted his left eye while gazing to the left. The maximum abduction he produced with his left eye is depicted (Figure 1). There were no Argyll Robertson pupils. Motor examination shows normal tone, bulk, and strength. There was no tremor, dystonia, myoclonus, or asterixis. There was normal sensation upon touching the patient. His gait was normal, and Romberg test was negative daily. Dysmetria and dysdiadochokinesia were not present during examination. CT and MRI of brain remained normal. Blood panel revealed a WBC as 3.9 × 10^3^/µL and platelet count of 179 × 10^3^/µL. Chemistry panel, renal function, and liver function were all within normal limits. The patients CD4 count is 80 cells/µL, and his HIV‐1 RNA quantitative reverse transcription PCR revealed a viral load of 110 000 copies/mL. His CSF analysis showed a WBC of 21 cell/cubic mm, 98% lymph. Protein was 49 mg/dL, and glucose was 29 mg/dL. There were only 2 red blood cells, and fluid was clear. CSF VDRL is 1:16. Other CSF studies (PCR HSV, West Nile, toxoplasmosis, cryptococcus, coccidioides, and JC Virus) were all negative. Blood RPR this time was 1:32.

A 2‐week course of penicillin G 3 million unit IV Q4 was initiated. Infectious disease team has decided to resume HAART only after the discharge. Daily neurological examination reveals partial improvement of his CN‐6 palsy by time of discharge.

A three‐month postdischarge phone follow‐up reveals resolution of diplopia, but patient had apparently refused a follow‐up for spinal tap. Thus, VDRL titers were not available as a follow‐up.

## 3 DISCUSSION

Syphilis, known as the great mimicker, has a very wide range of clinical presentation. Current cases only confirm that varying clinical manifestation of neurosyphilis can be challenging when trying to make a timely diagnosis and treatment. Furthermore, early neurosyphilis among HIV‐positive patients can be misdiagnosed by a clinician not thinking broadly about another coinfection.^8^

Due to the increased prevalence, there is a shift in focus on how neurosyphilis is diagnosed and treated.^9^, ^11^ Centers of Disease Control and Prevention provide a general criterion for classifications of neurosyphilis: neuropathic meningovascular, myelopathy, optical involvement including interstitial keratitis and uveitis, general paresis, including dementia, and tabes dorsalis. Due to various manifestations of neurosyphilis, a strong clinical judgment as well as laboratory testing is required to reduce diagnostic omission.^12^, ^13^, ^14^ No one‐laboratory assay has been proven to be sensitive enough to diagnose the disease.^15^, ^16^ Standard diagnostics involve nontreponemal screening followed by CSF nontreponemal‐specific antibody confirmatory tests.^17^ However, false‐positive and false‐negative results are common. A negative test may not necessarily exclude the diagnosis and vice versa; thus, a strong clinical judgment is necessary.^10^, ^15^ Hence, all patients suspected to be HIV positive with neurological presentation should undergo lumbar puncture with syphilis analysis. In addition, prompt diagnosis and combined penicillin‐corticosteroid therapy may improve neurological prognosis in early neurosyphilis with cranial nerve palsy.^18^, ^19^, ^20^

## CONFLICT OF INTEREST

None declared.

## AUTHOR CONTRIBUTIONS

SS: main author under AL's supervision. PP: secondary author. EL: resident who worked on the article. MM: resident who worked on the article. AL: attending neurologist and principal investigator.
